# Success of sky-polarimetric Viking navigation: revealing the chance Viking sailors could reach Greenland from Norway

**DOI:** 10.1098/rsos.172187

**Published:** 2018-04-04

**Authors:** Dénes Száz, Gábor Horváth

**Affiliations:** Environmental Optics Laboratory, Department of Biological Physics, ELTE Eötvös Loránd University, Pázmány sétány 1, 1117 Budapest, Hungary

**Keywords:** Viking navigation, sky polarization, sunstone, calcite, cordierite, tourmaline

## Abstract

According to a famous hypothesis, Viking sailors could navigate along the latitude between Norway and Greenland by means of sky polarization in cloudy weather using a sun compass and sunstone crystals. Using data measured in earlier atmospheric optical and psychophysical experiments, here we determine the success rate of this sky-polarimetric Viking navigation. Simulating 1000 voyages between Norway and Greenland with varying cloudiness at summer solstice and spring equinox, we revealed the chance with which Viking sailors could reach Greenland under the varying weather conditions of a 3-week-long journey as a function of the navigation periodicity Δ*t* if they analysed sky polarization with calcite, cordierite or tourmaline sunstones. Examples of voyage routes are also presented. Our results show that the sky-polarimetric navigation is surprisingly successful on both days of the spring equinox and summer solstice even under cloudy conditions if the navigator determined the north direction periodically at least once in every 3 h, independently of the type of sunstone used for the analysis of sky polarization. This explains why the Vikings could rule the Atlantic Ocean for 300 years and could reach North America without a magnetic compass. Our findings suggest that it is not only the navigation periodicity in itself that is important for higher navigation success rates, but also the distribution of times when the navigation procedure carried out is as symmetrical as possible with respect to the time point of real noon.

## 1. Introduction

Although Vikings did not have a magnetic compass, they had ruled the northern Atlantic Ocean for three centuries between about AD 900 and 1200 [1–5]. Their sailing success is attributed to the use of a genuine sun compass [3] with which they could determine the geographic north direction [6,7]. When the sun was hidden by clouds or thick fog, the navigator had to determine first the position of the invisible sun. According to the hypothesis of Ramskou [8], this was performed by means of skylight polarization and sunstone (e.g. calcite, cordierite or tourmaline) crystals functioning as linear polarizers. This method is the so-called sky-polarimetric Viking navigation, the atmospheric optical prerequisites of which have been intensely studied [9–17]. The uncertainties of the four main steps of this navigation method have been measured on test persons in psychophysical experiments in a laboratory and a planetarium [18–22].

Using the measured uncertainties of the four navigational steps, Száz *et al*. [21] determined the accuracy of sky-polarimetric navigation for 1080 different celestial situations characterized by the solar elevation angle *θ* and the cloudiness *ρ* (in oktas = 1/8 units), the sky polarization patterns of which have been measured by full-sky imaging polarimetry. As a result, they obtained the average ± standard error of the angular deviation from the north direction for the 1080 different sky situations (i.e. elevation *θ* and cloudiness *ρ* pairs) in the forenoon and afternoon at summer solstice and spring equinox for calcite, cordierite and tourmaline sunstone crystals.

In this work, we combine these previous results with computer simulations of navigated passages, to determine the likelihood that Viking voyages would successfully reach their intended destination, Greenland, under varying conditions of cloudiness during their voyage. We have simulated 1000 three-week-long Viking voyages along the latitude 60°21′55″ N from Norway to Greenland with randomly changing cloudiness at summer solstice and spring equinox, assuming that sky polarization was analysed by calcite, cordierite or tourmaline sunstones with different navigation periodicities Δ*t* = 1–6 h. A voyage route was considered successful if it approached the Greenland coastline within a critical distance, from which the mountains of Greenland can be seen, otherwise the route was unsuccessful. Finally, we obtained the per cent success of sky-polarimetric navigation at spring equinox and summer solstice for the three different sunstone crystals. To date, this is the most detailed and precise rating of sky-polarimetric navigation that is achievable without testing this method directly on the high seas.

## 2. Methods

### 2.1. Simulation of Viking sailing routes

In this work, we performed a computer simulation to follow the sailing routes of 1000 Viking voyages starting from Hernam (nowadays the Norwegian Bergen) along the 60°21′55″ N latitude, which was the main sailing route of the Vikings to their settlement Hvarf in south Greenland. For computing geographical distances, we used a map with planar representation (Mercator projection) with the vertical lines of longitude being perpendicular to the horizontal lines of latitude. This well-known method has a distortion problem only near the North and the South Poles; however, at the 60°21′55″ N latitude this problem was practically negligible.

The maps in figures 1 and 3 and electronic supplementary material, figures S1–S36 were generated by a software written by us. The contours of continents and islands were manually digitalized from the map available as open-source data from http://www.gnuplotting.org/plotting-the-world-revisited/ (the raw data points of the contours can be freely downloaded in text format from: http://www.gnuplotting.org/data/world_10m.txt). These open-source data can be freely used without permission/licence.

**Figure 1.**
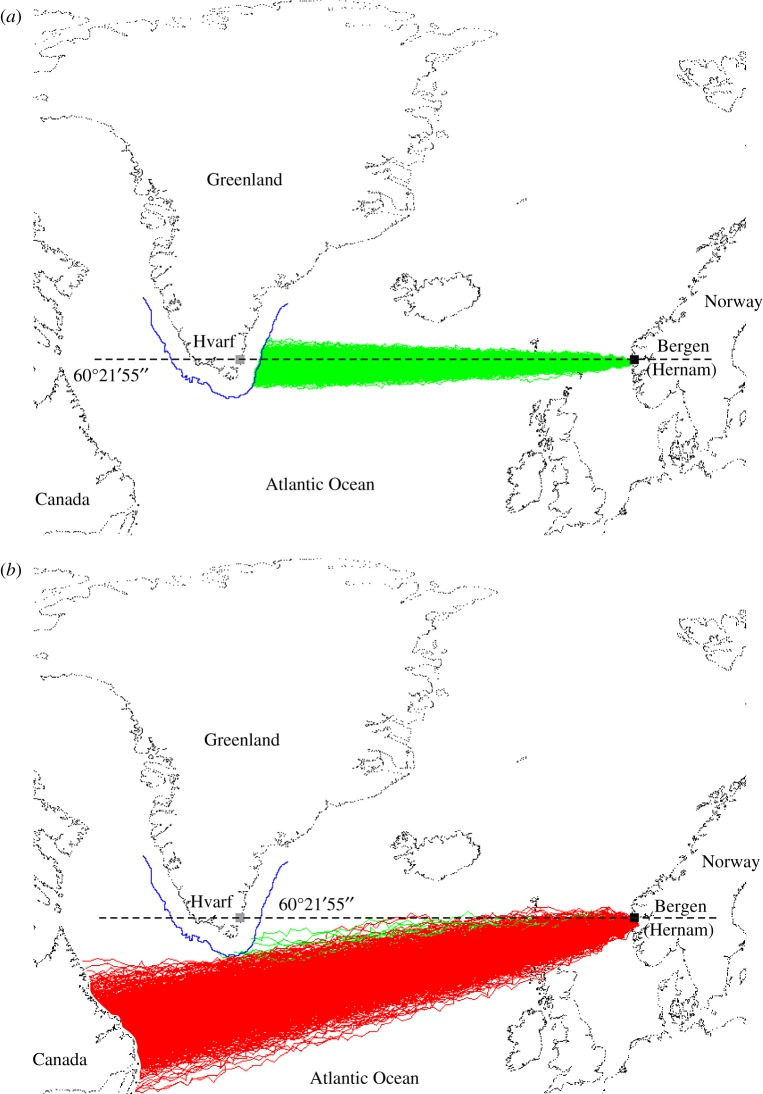
Simulated Viking sailing routes from Norway to Greenland. Successful (green) and unsuccessful (red) routes of 1000 Viking voyages from Bergen (Norway) to Greenland at spring equinox, if a calcite sunstone crystal is used to analyse sky polarization with a navigation periodicity Δ*t* = 1 h (*a*), and Δ*t* = 6 h (*b*), when the navigation success is *s* = 100% (*a*), and *s* = 0.9% (*b*) (electronic supplementary material, table S1). The blue curve is the borderline of visibility from which the southernmost mountains of Greenland can already be seen from a Viking ship. The map was generated by our self-written software after a manual selection of the contours of continents and islands from the open-source data available freely from http://www.gnuplotting.org/plotting-the-world-revisited/. In (*b*), some red sailing trajectories went through North Scotland. In these cases, it was assumed that the Vikings continued their voyage towards Greenland.

From Bergen to Greenland, the Viking sailors had to accurately keep the western direction parallel to the 60°21′55″ N latitude. Thus, a sailing route with no navigation uncertainty would be a straight horizontal line on our planar map. The navigation uncertainties cause the sailing route to deviate from the east–west direction more or less northwards or southwards depending on the sign (+/–) and the degree of the navigation uncertainty. Viking voyages were simulated for two specific dates, spring equinox (21 March) and summer solstice (21 June), to which the hourly solar elevation angles for the 60°21′55″ N latitude were calculated with an online solar elevation calculator (https://www.sunearthtools.com/dp/tools/pos_sun.php). Thus, we obtained a database for the hourly solar elevation angle on the 60°21′55″ N latitude at summer solstice and spring equinox. The speed of the Viking ships was set to 11 km h^−1^. The Vikings used different types of ships with different maximal velocities, and we selected the average speed of an average-sized ship with a mast height *h* = 21 m [5]. The sailing routes were calculated with a self-written computer program, the algorithm of which was the following:

(1) A Viking navigator started to navigate in the morning (at sunrise) of a given date, when the sun elevation angle *θ* was 0° from the horizon.
(2) To determine the navigation uncertainty (=angle of deviation from the geographical north), first we randomly chose a value of cloudiness *ρ* from 0 to 8 oktas with a uniform distribution. Then, we used the 1080 raw data files from the *θ*–*ρ* matrix determined by Száz *et al*. [21]. In this matrix, each *θ*−*ρ* pair contained 12 different sky situations with a known navigation uncertainty distribution calculated with the use of the uncertainties of the four steps of sky-polarimetric Viking navigation measured in psychophysical laboratory/planetarium experiments. One of these 12 cloudy sky situations was selected randomly according to uniform distribution, then from the corresponding uncertainty distribution we used a randomly chosen uncertainty value as the navigation uncertainty.
(3) The ship moved in the resulting direction along a straight line with a 11 km h^−1^ velocity until the navigator determined again the sailing direction to be followed after a possibly constant time period *τ*. As the Vikings did not have a timepiece to measure time accurately, they had to rely on their subjective time sensation. Therefore, *τ* was not a constant, but had also an uncertainty which was approximated with *Δt*/6 in our simulations, where Δ*t* is called the appointed navigation periodicity, being equal to the elapsed time since the last navigation. Here, *τ* was chosen randomly according to a uniform distribution from the range Δ*t* – Δ*t*/6 ≤ *τ* ≤ Δ*t* + Δ*t*/6. Hence, we assumed that the navigator wanted to determine the sailing direction with a constant appointed periodicity Δ*t*, but he could perform this only with a randomly changing semi-periodicity *τ*, the maximal uncertainty of which (Δ*t*/6) was proportional to the appointed Δ*t*.
(4) For each new navigation, knowing *τ*, we determined the current solar elevation angle *θ* from the database (https://www.sunearthtools.com/dp/tools/pos_sun.php). The new cloudiness *ρ* was computed in the following way: The change of oktas Δ*ρ* was obtained by choosing a Gaussian-weighted random number from the range –8 ≤ Δ*ρ* ≤ +8 with an expected value 0 and standard deviation 2. This means that small changes in the cloudiness *ρ* within a short time have larger possibilities than big ones. The new cloudiness was *ρ* + Δ*ρ* with the condition that it must be between 0 and 8 oktas. To fulfil this condition, the new cloudiness was set to 0 when *ρ* + Δ*ρ* < 0, and was set to 8 when *ρ* + Δ*ρ* > 8. For intermediate cases, the obtained values of *ρ* + Δ*ρ* were used. With the new *θ*–*ρ* parameters, we randomly selected a sky situation from the corresponding 12 ones.
(5) Steps 3–4 were repeated until the solar elevation angle became negative, that is the sun was below the horizon. After sunset, the Vikings were assumed to stop their ships and did not move until the next sunrise. Between sunset and sunrise, the ship's position was assumed to be constant (the random drift due to winds and water currents was neglected).
(6) Steps 1–5 were repeated until the ship reached the borderline of visibility of the mountains of Greenland (successful sailing route), or got too far from this borderline, getting lost in the ocean (unsuccessful sailing route).
(7) The above computations were performed 1000 times for three sunstone crystals (calcite, cordierite and tourmaline), two dates (spring equinox and summer solstice) and six navigation periodicities Δ*t* (=1, 2, 3, 4, 5, 6 h). Thus altogether we obtained 36 000 simulated sailing routes.

### 2.2. Determination of the navigation success

To determine the success of a voyage from Bergen to Greenland, we defined the borderline near the coastline of Greenland from where the navigator could see the mountains of Greenland. The distance of this border from the coast was calculated as follows (electronic supplementary material, figure S37): (i) the average height of the mountains of Greenland is *m* = 1000 m; the first mountains are on average at distance *c* = 1000 m from the coast inside the island. A Viking observer could climb up on the mast of a Viking ship up to a height *h* = 21 m (above sea level) in order to see the mountains of Greenland as soon as possible. (ii) The radius of the Earth is *r* = 6372.8 km, and the Earth's shape was approximated by a sphere. (iii) The distance from where the top of the mountains can already be seen is *d* = *r*(*α* + *β*) − *c*, where *α* = arc cos[*r*/(*r*  +  *m*)] is the angular distance where the tangential straight line from the top of the mountain reaches the Earth's surface, and *β* = arc cos[*r*/(*r* + *h*)] is the angular distance measured from *α* where this tangential line reaches the observer at a height *h* on the ship's mast (electronic supplementary material, figure S37).

A voyage was considered as successful if the simulated sailing route crossed the borderline of visibility of the mountains of Greenland, otherwise it was unsuccessful. For a given sunstone crystal (calcite, cordierite and tourmaline), a given date (spring equinox and summer solstice) and a given navigation periodicity Δ*t* (=1, 2, 3, 4, 5, 6 h) we simulated *N* = 1000 voyages, from which *N*_s_ was successful and *N*_u_ was unsuccessful (*N* = *N*_s_ + *N*_u_). Finally, we computed the navigation success *s* = *N*_s_/*N* in all 36 cases = 3 (sunstones) × 2 (dates) × 6 (navigation periodicities).

We also simulated reversed voyages from Hvarf (Greenland) to Norway along the 60°21′55″ N latitude. However, in these cases the voyages were always successful, because the simulated sailing routes always reached somewhere on the coasts of Europe.

## 3. Results

Figure 1 shows two extreme cases of 1000 simulated Viking voyages from Bergen (Norway) to Greenland at spring equinox if a calcite sunstone crystal is used to analyse sky polarization. In figure 1*a,* the navigation periodicity is Δ*t* = 1 h and all the 1000 sailing routes are successful (marked with green), because all reach the coast of Greenland. If, however, Δ*t* = 6 h (figure 1*b*), among the 1000 routes only 9 are successful (green) and 991 are unsuccessful (red) because the latter never reach Greenland.

Figure 2 shows (and electronic supplementary material, table S1 contains) the navigation success *s* of sky-polarimetric navigation between Bergen and Greenland at spring equinox and summer solstice for calcite, cordierite and tourmaline sunstone crystals if the navigation periodicity is Δ*t* = 1, 2, 3, 4, 5 and 6 h. Electronic supplementary material, figures S1–S36 display the successful (green) and unsuccessful (red) sailing routes in all these 2 × 3 × 6 = 36 cases.

**Figure 2.**
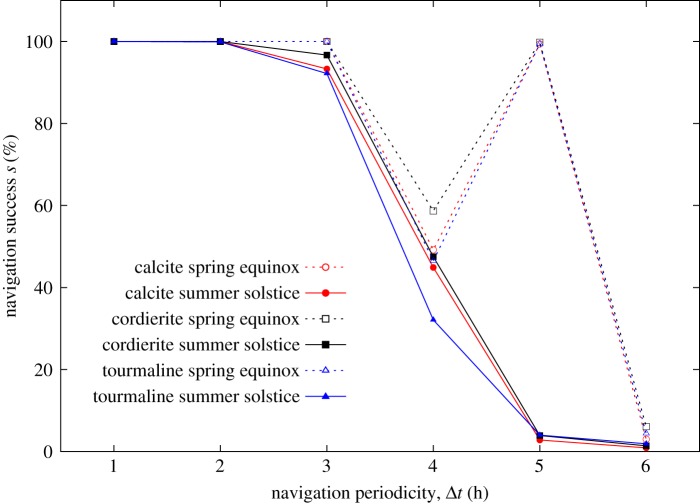
Success rate of Viking voyages. Navigation success *s* (%) of sky-polarimetric Viking navigation on the voyage between Bergen (Norway) and Greenland at spring equinox and summer solstice for calcite, cordierite and tourmaline sunstone crystals as a function of the navigation periodicity Δ*t* (h) (electronic supplementary material, table S1).

If the navigation periodicity Δ*t* is 1, 2 or 3 h, then the navigation success *s* is between 92.2 and 100% for all three sunstones and at both spring equinox and summer solstice. For Δ*t* = 4 h, however, only 32.1–58.7% of all voyages are successful at both dates. For Δ*t* = 5 h, the navigation success *s* is lower than 4% at summer solstice for all sunstone crystals, but surprisingly, at spring equinox *s* approximates 100% for all sunstones, the reason of which is in the daily (a)symmetry of navigation times (see Discussion). For Δ*t* = 6 h, the navigation success drops to 3.0–6.1% and 0.9–1.9% at spring equinox and summer solstice, respectively.

From these results we conclude that the sky-polarimetric Viking navigation is surprisingly successful at spring equinox and summer solstice even in cloudy weather if the navigator determines the sailing (west) direction periodically at least once in every 3 h, independently of the type of sunstone (calcite, cordierite, tourmaline) used for the analysis of sky polarization. It is noteworthy, that for Δ*t* = 1–4 h, cordierite ensures the most accurate navigation (highest navigation success *s*) and tourmaline results in the most erroneous one (lowest *s*), while for Δ*t* = 6 h, calcite is the worst sunstone with the lowest *s*-values.

**Figure 3.**
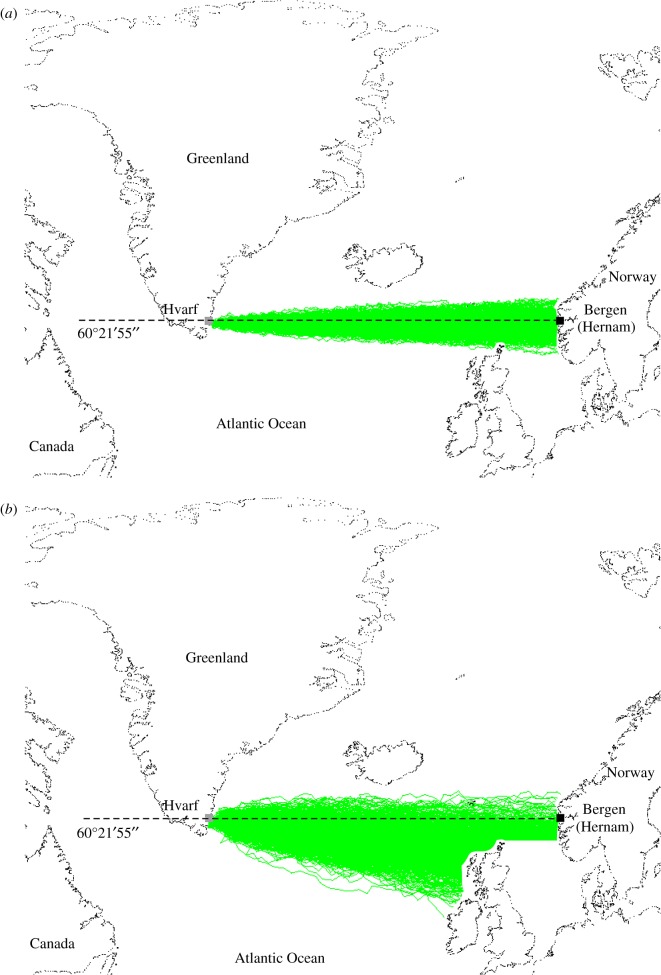
Simulated Viking sailing routes from Greenland to Norway. Simulated (successful: green) routes of 1000 Viking voyages from Hvarf (Greenland) to Norway at spring equinox, if a calcite sunstone crystal is used to analyse sky polarization with a navigation periodicity (*a*) Δ*t* = 1 h, and (*b*) Δ*t* = 6 h.

Figure 3 shows 1000 simulated Viking sailing routes from Hvarf (Greenland) to Norway at spring equinox if a calcite sunstone crystal is used to analyse sky polarization with a navigation periodicity Δ*t* = 1 and 6 h. All these voyages are successful, because they terminate somewhere on the coasts of Europe.

## 4. Discussion

In this work, we concentrated on the determination of the success of sky-polarimetric Viking navigation along the 60°21′55″ northern latitude, the main sailing route of the Vikings between Norway and Greenland. We obtained surprisingly large success rates of this navigation method in cloudy weather.

We studied thoroughly Viking voyages from Norway to Greenland only, because the reverse voyages from Greenland to Norway were less critical, because they terminated necessarily somewhere on the coasts of the European continent if Viking sailors had enough drinking (fresh) water and food and did not sink. This is demonstrated in figure 3.

In certain cases, many simulated sailing routes tend more or less southwards (electronic supplementary material, figures S4, S6, S10, S12, S16, S18, S21–S24, S28–S30, S33–S36) or northwards (electronic supplementary material, figures S1–S3, S7–S9, S11, S13–S15). The reason for this is the following: As the sign of navigation uncertainties (i.e. angles of deviation from the geographical north) is overwhelmingly plus (+) in the forenoon and minus (–) in the afternoon, the sailing routes tend northwards and southwards in the forenoon and afternoon, respectively. This is the reason for the zigzagging of the sailing routes (figures 1 and 3, electronic supplementary material, figures S1–S36). We assumed that during a voyage the Viking navigator determined the north direction (and from this the west direction to be followed) after every *Δt* period between sunrise and sunset. If the number *n*_f_ of navigation in the forenoon and the number *n*_a_ of navigation in the afternoon are the same (*n*_f_ = *n*_a_), the net northward and southward deviations of the sailing route are approximately the same, and thus the average sailing route is nearly straight along the 60°21′55″ latitude. If, however, *n*_f_ > *n*_a_ due to specific Δ*t*-values, then northward deviations dominate and the route tends northwards, while if *n*_f_ < *n*_a_, then the southward deviations dominate and the route tends southwards. Northward-tending routes occur more times at spring equinox than at summer solstice, while southward deviation characterized the routes at summer solstice. A navigation day (from sunrise to sunset) lasted only for 12 h at the equinox and 17 h at the solstice on the 60°21′55″ latitude.

The same effect results in the sudden drops in navigation success *s* with increasing navigation periodicity Δ*t* (figure 2; electronic supplementary material, table S1): For Δ*t* = 1, 2 or 3 h, *n*_f_ is approximately the same as *n*_a_, thus the sailing routes cannot tend significantly southwards or northwards. Consequently, these routes can easily reach Greenland, and thus *s* is very high, independently of the date (spring equinox or summer solstice). However, for Δ*t* = 4, 5 or 6 h, *n*_f_ can be much smaller than *n*_a_, thus the sailing routes can tend so strongly southwards that they never reach Greenland, and therefore *s* can decrease significantly, depending on the date. *n*_f_ can also be much larger than *n*_a_, when the sailing routes can tend strongly northwards, but in this case they necessarily reach Greenland, and therefore *s* does not drop, independently of the date. This also explains why the navigation success rate is so large (approx. 100%) at spring equinox for Δ*t* = 5 h, where the distribution of the routes is large; however, they mainly tend northwards. This temporal interference of forenoon and afternoon with the navigation periodicity Δ*t* is the main reason for the sudden drops in navigation success *s* with increasing Δ*t* (figure 2; electronic supplementary material, table S1).

Our findings suggest that it is not only the navigation periodicity Δ*t* in itself that is important for higher navigation success rates, but also the distribution of times when the navigation procedure carried out is as symmetrical as possible with respect to the time point of real noon.

In cases when the sailing routes tended considerably southwards, Viking voyages never reached Greenland, but terminated with death of the whole crew in the Atlantic Ocean, or reached North America (now Canada) (figure 1*b*). The latter might have resulted in the accidental discovery of America by the Vikings much earlier than Columbus. There is archaeological evidence of Viking settlements along the western coastline of Newfoundland (Canada) [2,3].

It is important to note that we slightly overestimated the navigation success, because it was assumed that during the voyage there were no strong winds or storms that could interrupt the voyage to Greenland; furthermore, we assumed that Viking ships did not drift away at night, when the sailing was stopped. Owing to the random combination of clear and cloudy skies and the above assumptions, the success of Viking voyages from Norway to Greenland might have been somewhat lower than obtained in this work for sky-polarimetric navigation (electronic supplementary material, table S1; figure 2).

In the simulation of sailing routes, we did not take into consideration the influence of water currents and wind, because these would have diverted the simulated Viking ships northwards and thus would have increased the success rate of their journey. The average speed of water in the Atlantic Ocean is 15–20 cm s^−1^ = 0.54–0.72 km h^−1^ and it is heading northeast [23]. The average wind speed above the Atlantic Ocean is much larger—50–60 km h^−1^ [23]—but over the North Atlantic prevailing winds blow towards west or southwest [23]. Sailing direction deviations suffered on the dangerous waters of Cape Farewell by the coast of Greenland could not be considered as a systematic effect. Wind conditions during the Viking era could differ from those of nowadays, but we consider that they could partly compensate for the uncertainties of sky-polarimetric navigation.

It was a logical assumption that between sunset and sunrise the Vikings lowered their sails and thus stopped their voyage, because until the stars (e.g. the Polaris) were visible, they could not navigate. With an unlowered sail the ship would have randomly drifted due to the random winds, which would have made the safe continuation of the voyage difficult. With a lowered sail the ship drifted away only slightly at night, thus its position practically did not change much, as we assumed in the simulation.

Vikings have also used some supporting and mitigating navigational methods, such as wave direction, solar midday elevation, erring to the north, proximity of the Faroes or Shetland Isles, for instance [3,5]. These might have improved the overall navigational reliability, and perhaps alleviated some of the difficulties that would have been encountered, too.

We simulated 100, 500 and 1000 Viking sailing routes for the six different situations (equinox +solstice, calcite + cordierite + tourmaline sunstone crystals), and experienced that the navigation successes converged towards the values presented in this work (obtained for 1000 routes). For comparison, we also performed 10 000 sailing route simulations for some parameter settings, but did not obtain significant differences in the success rate compared to the corresponding 1000 simulations; only the computation time increased 10-fold. From this, we concluded that simulating 1000 routes was enough (for a given set of conditions), that is, it sufficiently approximated the case of simulating much larger (infinite) route numbers and the computation time could be kept optimally low.

Let us estimate the realistic total number of routes of a Viking ship during the nearly 300 years of the Viking era: The duration of a complete route from Norway to Greenland and back to Norway could be a minimum of 6 weeks. The sailing period might have been between April and September (when the Atlantic Ocean was iceless along the 61° latitude), meaning yearly 6 × 4 = 24 weeks, during which four complete to-and-back routes could be done with a given ship. Thus, during 300 years, a given ship could perform 4 × 300 = 1200 complete routes between Norway and Greenland. Owing to ship amortization and bad weather conditions this is an overestimated number. More realistic is about 1000 complete routes for a ship, if it was always more or less repaired after every route.

During a pilot simulation we have varied the constant velocity *v* of the Viking ship, the typical value of which was 11 km h^−1^ [5]. We obtained the trivial result that a decrease/increase of *v* resulted in only the increase/decrease of the time after which the simulated ship reached Greenland from Norway without any significant change in the navigation success. In future studies, the ship velocity *v* could be varied, either with a random distribution or according to a function of cloudiness or a distribution that is not symmetric with regard to forenoons and afternoons. However, according to our pilot simulations, we do not expect significant changes of the navigation success from the randomization of *v*. On the one hand, an asymmetry of the distribution of *v*-values relative to forenoons and afternoons could trivially increase or decrease the navigation success, if the forenoon or afternoon *v*-values were tendentiously greater, because the sailing routes would tendentiously deviate northwards (helping to reach some place on the coastline of Greenland) or southwards (failing to reach Greenland); on the other hand, we do not know any physical/meteorological phenomenon that could result in such an asymmetry of *v*.

In our simulations, we assumed that the visibility of the coastline of Greenland is good and constant. The calculated borderline--coastline distance used by us (electronic supplementary material, figure S37) is valid only for a clear atmosphere. It can be reduced up to zero in fog or heavy rain. If there were low visibility conditions in the vicinity of the Greenland shore (so that mountains could not be seen even though in theory the curvature of the Earth allows for it), the navigation success would obviously decrease. In other words, this would represent a hazard for the successful arrival of Viking sailors relying on sky-polarimetric navigation. Note that the calculation of dipping distances is a standard component of visual maritime navigation (albeit rarely tabulated for objects as high as mountains), and in determining the elevations of heavenly bodies, atmospheric refraction is also taken into account. We neglected the latter atmospheric optical effect in our simulations.

As a logical continuation of our present simulations, in the future it would also be interesting to perform the following studies:

— How would the navigation success change if strong changes in cloudiness within small time period(s) were allowed, as opposed to the more conservative setting used in this work?
— If some real-world data regarding the variation of cloudiness during a day, in various seasons of the year in the relevant geographic region (along the 61° latitude) would be accessible (presently we do not know such data source), new simulations could be done. However, we expect that with such a study only the trivial result could be obtained that the more frequently strongly cloudy skies occur, the larger is the decrease in navigation success.
— It would also be interesting to know (i) the navigational uncertainty that results from a given (e.g. ±1°) error in the sunstone crystal marking or (ii) how the change of the (constant or randomized) phase of the first navigation time relative to sunrise influences the navigation success. Note that in our simulations this phase was always zero, that is the first navigation procedure (i.e. determination of the intended sailing direction) happened immediately after sunrise. It is logical to assume that Vikings set sail immediately at sunrise when they had the opportunity to continue their journey. The effect of consistent overestimations/underestimations of the deduced solar elevation was measured by Száz *et al*. [20] and was included in the computation of the *θ−ρ* matrix of the north uncertainties [21].
— Further simulations could also answer the question: How robust are the very high navigation success rates obtained, with respect to altering various parameters and environmental/meteorological conditions? The aim of such an approach could be to find those parameter settings which result in a breakdown of the success rates. Our present paper explicitly shows such an example for navigation periodicities Δ*t* = 4, 5 and 6 h (figure 2), but it would also be interesting to check other parameters. If it could be consistently shown that the breakdown of successful navigation only occurs for simulated conditions that are far from (or rare in) reality, then this would well demonstrate the robustness of our findings presented here.

The famous theory/hypothesis of sky-polarimetric Viking navigation [8] is frequently cited [3,5,9–22,24–31]. Now, we showed that this navigation method can function well under cloudy skies on a voyage with varying cloudiness if the navigation periodicity Δ*t* is small enough and is distributed symmetrically before and after the time point of the real noon. Nobody knows whether the Vikings really used this method. However, if they did, they could navigate with it precisely. Further studies (simulations) could decide whether the obtained high (90–100%) navigation success rates for small navigation periodicities Δ*t* = 1–3 h are robust enough under various environmental conditions.

## Supplementary Material

Supplementary Materials

## Data accessibility

Our paper has the following electronic supporting materials: electronic supplementary material, table S1 and figures S1–S37.
