# Supplementary material for: Success of sky-polarimetric Viking navigation: revealing the chance Viking sailors could reach Greenland from Norway
Source: R Soc Open Sci. 2018 Apr 4;5(4):172187. doi: 10.1098/rsos.172187 (PMC5936938; doi:10.1098/rsos.172187)

**Supplementary Materials**

for

**Success of sky-polarimetric Viking navigation: revealing the chance Viking sailors could reach Greenland from Norway**

Dénes Száz^1^ and Gábor Horváth^1,^*

^1^Environmental Optics Laboratory, Department of Biological Physics, ELTE Eötvös Loránd University, H-1117 Budapest, Pázmány sétány 1, Hungary

*Corresponding Author, e-mail address: gh@arago.elte.hu

Changes done on the basis of the review of Referee 1 are marked by blue

This file contains the following: Supplementary Table S1

Supplementary Figures S1 – S37

The map in Figs. 1, 3 and Supplementary Figs. S1-S36 was generated by a software written by us. The contours of continents and islands were manually digitalized from the map available as open-source data from http://www.gnuplotting.org/plotting-the-world-revisited/ (the raw data points of the contours can be freely downloaded in text format from: http://www.gnuplotting.org/data/world_10m.txt). These open-source data can be freely used without permission/licence.

**Supplementary Table S1.** Navigation success (%) of sky-polarimetric Viking navigation on the voyage between Bergen (Norway) and Greenland at spring equinox and summer solstice for calcite, cordierite and tourmaline sunstone crystals as a function of the navigation periodicity Δ*t* (hour) (Fig. 2).

| **sunstone**  **crystal** | **navigation periodicity Δ*t*** | | | | | |
| --- | --- | --- | --- | --- | --- | --- |
|  | **1 h** | | **2 h** | | **3 h** | |
|  | **spring**  **equinox** | **summer**  **solstice** | **spring**  **equinox** | **summer**  **solstice** | **spring**  **equinox** | **summer**  **solstice** |
| **calcite** | 100.0 | 100.0 | 100.0 | 100.0 | 100.0 | 93.3 |
| **cordierite** | 100.0 | 100.0 | 100.0 | 100.0 | 100.0 | 96.7 |
| **tourmaline** | 100.0 | 100.0 | 100.0 | 99.9 | 100.0 | 92.2 |
|  | **4 h** | | **5 h** | | **6 h** | |
| **calcite** | 49.2 | 44.9 | 99.4 | 2.8 | 3.0 | 0.9 |
| **cordierite** | 58.7 | 47.5 | 99.8 | 3.9 | 6.1 | 1.4 |
| **tourmaline** | 46.5 | 32.1 | 99.4 | 4.0 | 4.4 | 1.9 |

**Supplementary Figure S1.** Simulated successful (green) and unsuccessful (red) routes of 1000 Viking voyages from Bergen to Greenland at spring equinox, if a calcite sunstone crystal is used to analyse sky polarization with a navigation periodicity Δ*t* = 1 h, when the navigation success is 100 % (Supplementary Table S1).


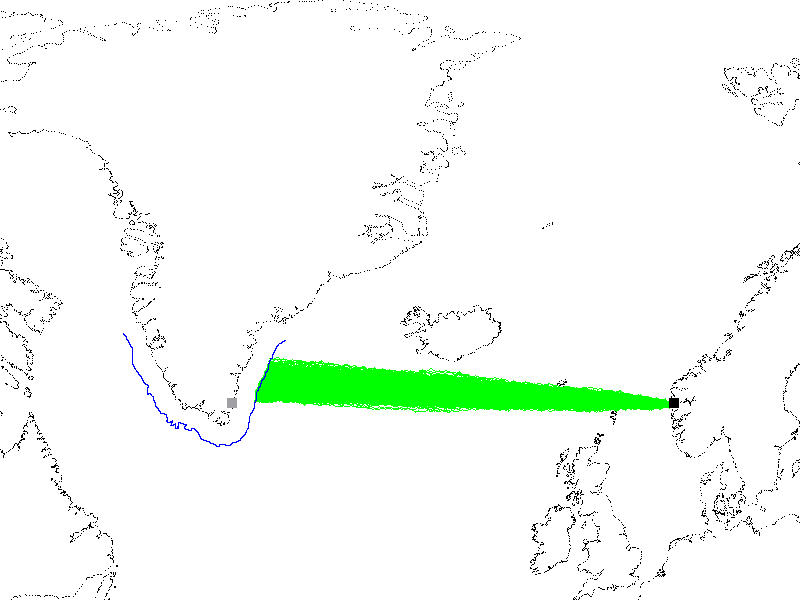


**Supplementary Figure S2.** Simulated successful (green) and unsuccessful (red) routes of 1000 Viking voyages from Bergen to Greenland at spring equinox, if a calcite sunstone crystal is used to analyse sky polarization with a navigation periodicity Δ*t* = 2 h, when the navigation success is 100 % (Supplementary Table S1).


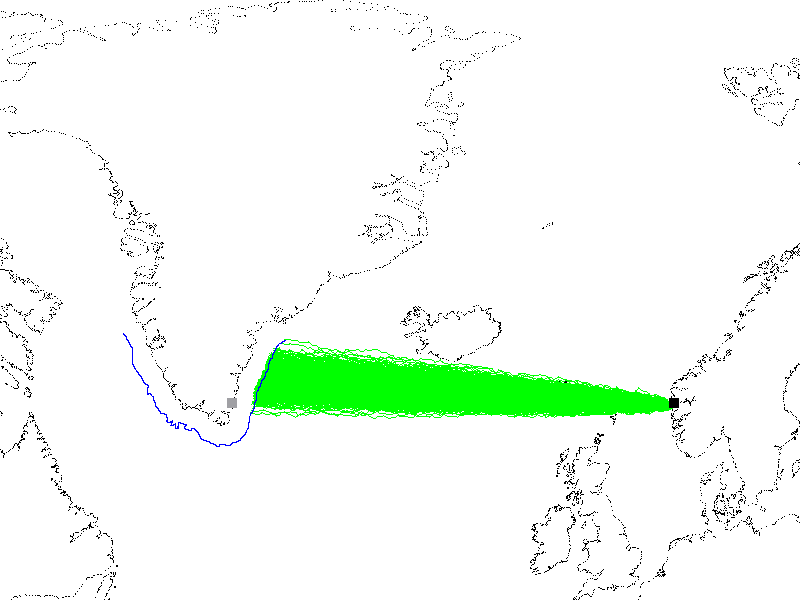


**Supplementary Figure S3.** Simulated successful (green) and unsuccessful (red) routes of 1000 Viking voyages from Bergen to Greenland at spring equinox, if a calcite sunstone crystal is used to analyse sky polarization with a navigation periodicity Δ*t* = 3 h, when the navigation success is 100 % (Supplementary Table S1).


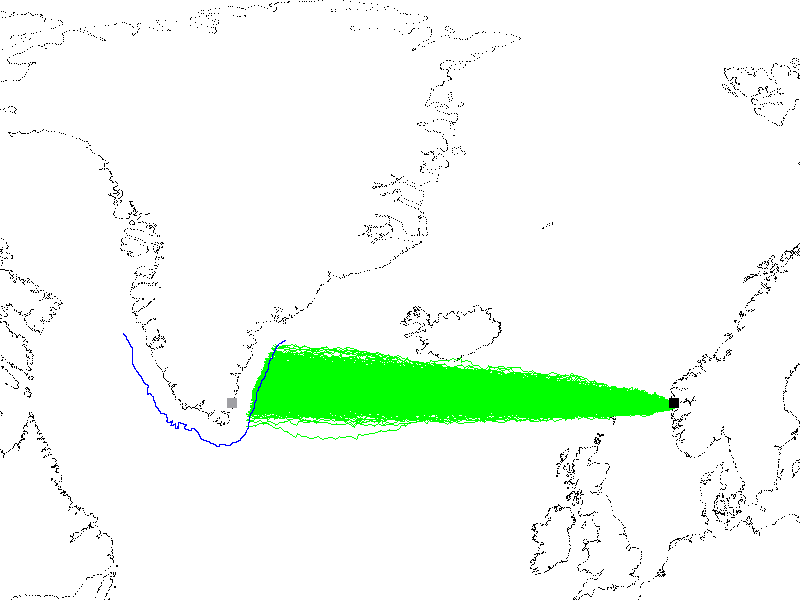


**Supplementary Figure S4.** Simulated successful (green) and unsuccessful (red) routes of 1000 Viking voyages from Bergen to Greenland at spring equinox, if a calcite sunstone crystal is used to analyse sky polarization with a navigation periodicity Δ*t* = 4 h, when the navigation success is 49.2 % (Supplementary Table S1).


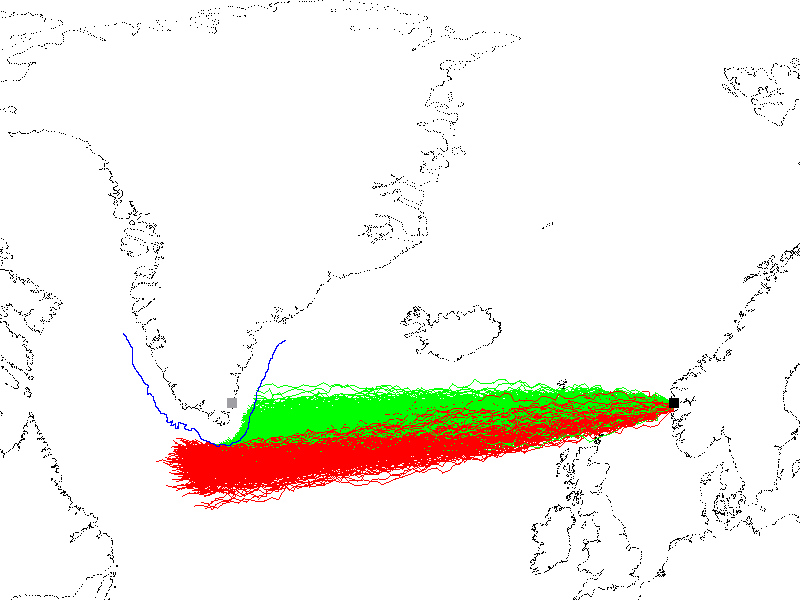


**Supplementary Figure S5.** Simulated successful (green) and unsuccessful (red) routes of 1000 Viking voyages from Bergen to Greenland at spring equinox, if a calcite sunstone crystal is used to analyse sky polarization with a navigation periodicity Δ*t* = 5 h, when the navigation success is 99.4 % (Supplementary Table S1). If some sailing trajectories went through Iceland, it was assumed that the Vikings continued their voyage toward Greenland.


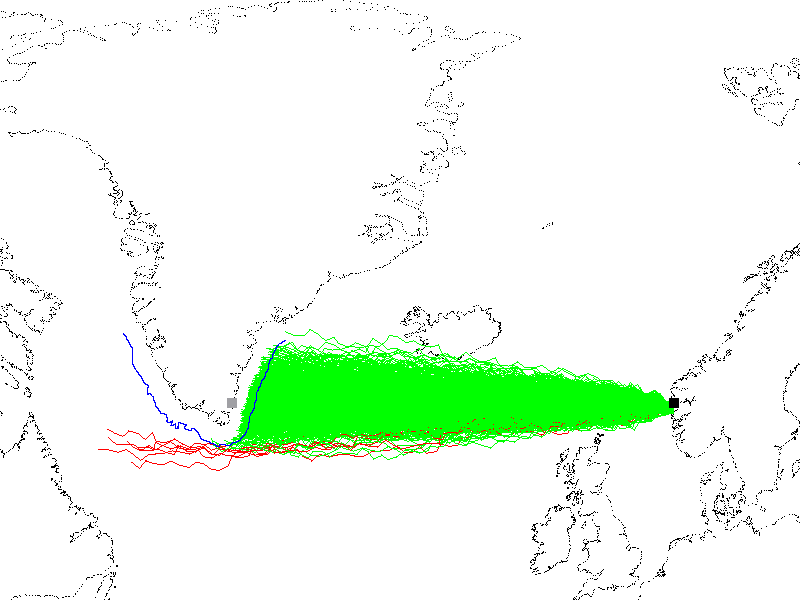


**Supplementary Figure S6.** Simulated successful (green) and unsuccessful (red) routes of 1000 Viking voyages from Bergen to Greenland at spring equinox, if a calcite sunstone crystal is used to analyse sky polarization with a navigation periodicity Δ*t* = 6 h, when the navigation success is 3 % (Supplementary Table S1). If some sailing trajectories went through North Scotland, it was assumed that the Vikings continued their voyage toward Greenland.


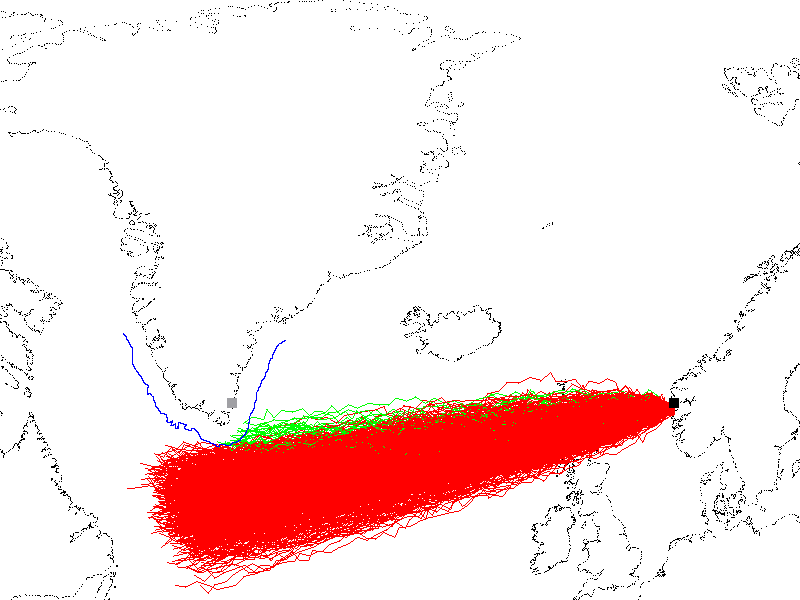


**Supplementary Figure S7.** Simulated successful (green) and unsuccessful (red) routes of 1000 Viking voyages from Bergen to Greenland at spring equinox, if a cordierite sunstone crystal is used to analyse sky polarization with a navigation periodicity Δ*t* = 1 h, when the navigation success is 100 % (Supplementary Table S1).


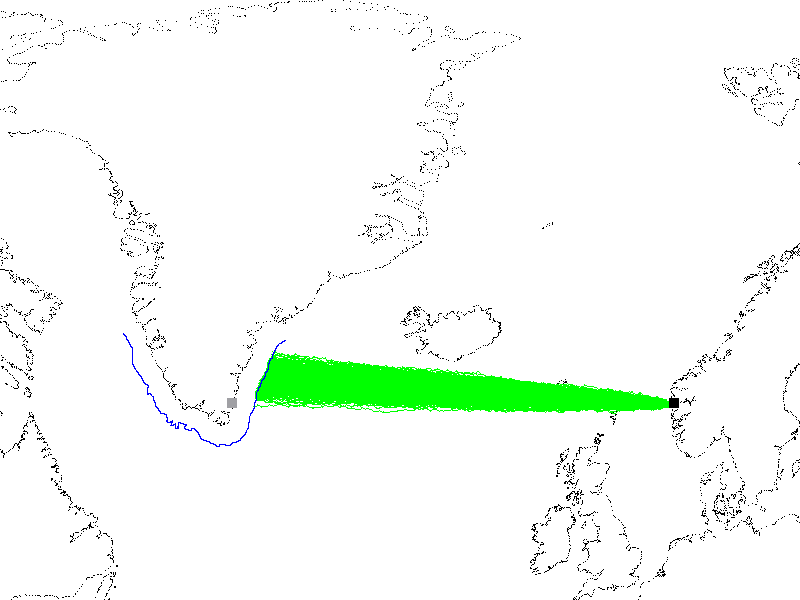


**Supplementary Figure S8.** Simulated successful (green) and unsuccessful (red) routes of 1000 Viking voyages from Bergen to Greenland at spring equinox, if a cordierite sunstone crystal is used to analyse sky polarization with a navigation periodicity Δ*t* = 2 h, when the navigation success is 100 % (Supplementary Table S1). If some sailing trajectories went through Iceland, it was assumed that the Vikings continued their voyage toward Greenland.


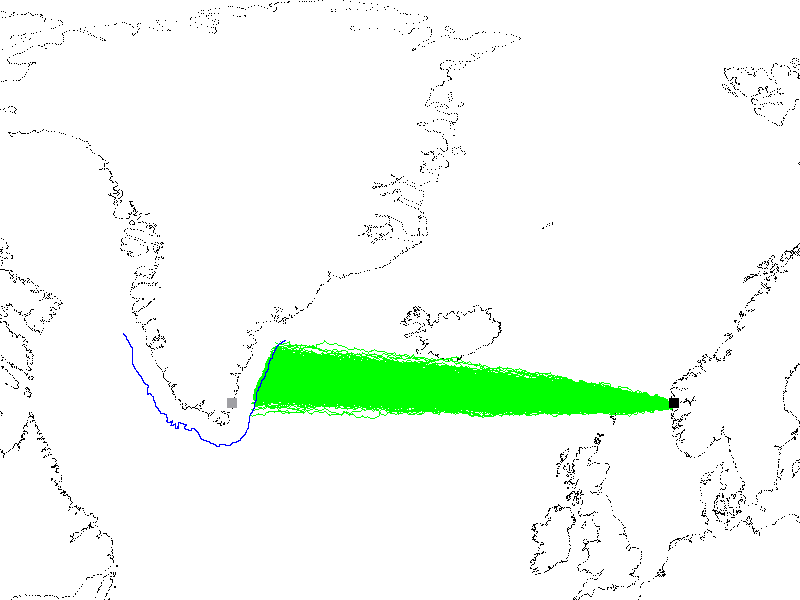


**Supplementary Figure S9.** Simulated successful (green) and unsuccessful (red) routes of 1000 Viking voyages from Bergen to Greenland at spring equinox, if a cordierite sunstone crystal is used to analyse sky polarization with a navigation periodicity Δ*t* = 3 h, when the navigation success is 100 % (Supplementary Table S1). If some sailing trajectories went through Iceland, it was assumed that the Vikings continued their voyage toward Greenland.


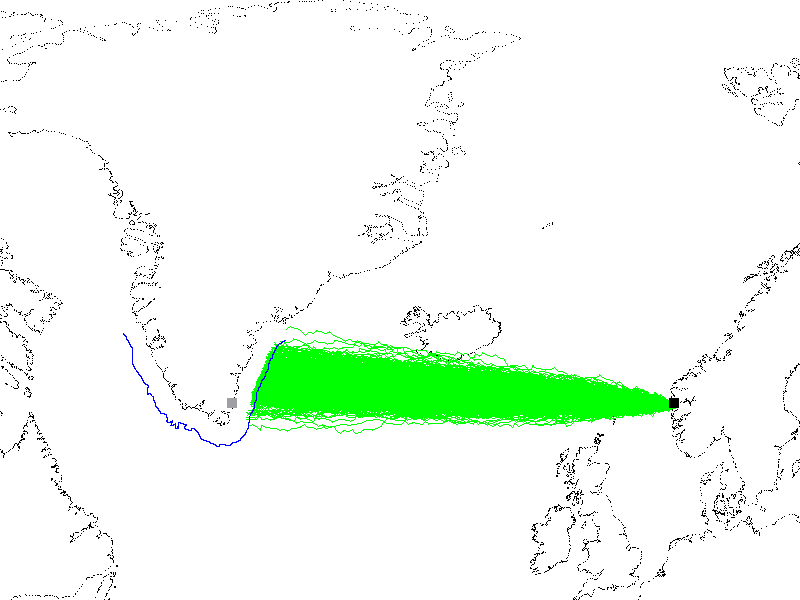


**Supplementary Figure S10.** Simulated successful (green) and unsuccessful (red) routes of 1000 Viking voyages from Bergen to Greenland at spring equinox, if a cordierite sunstone crystal is used to analyse sky polarization with a navigation periodicity Δ*t* = 4 h, when the navigation success is 58.7 % (Supplementary Table S1).


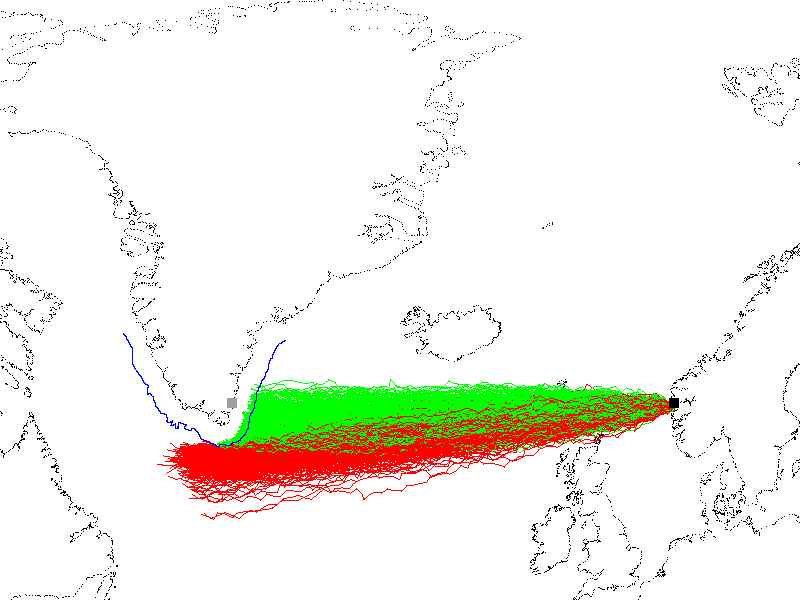


**Supplementary Figure S11.** Simulated successful (green) and unsuccessful (red) routes of 1000 Viking voyages from Bergen to Greenland at spring equinox, if a cordierite sunstone crystal is used to analyse sky polarization with a navigation periodicity Δ*t* = 5 h, when the navigation success is 99.8 % (Supplementary Table S1). If some sailing trajectories went through Iceland, it was assumed that the Vikings continued their voyage toward Greenland.


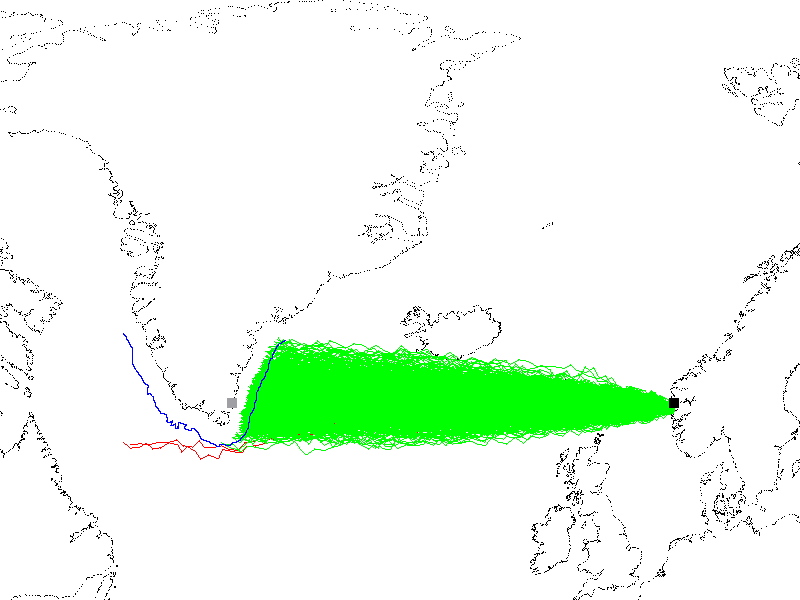


**Supplementary Figure S12.** Simulated successful (green) and unsuccessful (red) routes of 1000 Viking voyages from Bergen to Greenland at spring equinox, if a cordierite sunstone crystal is used to analyse sky polarization with a navigation periodicity Δ*t* = 6 h, when the navigation success is 6.1 % (Supplementary Table S1). If some sailing trajectories went through North Scotland, it was assumed that the Vikings continued their voyage toward Greenland.


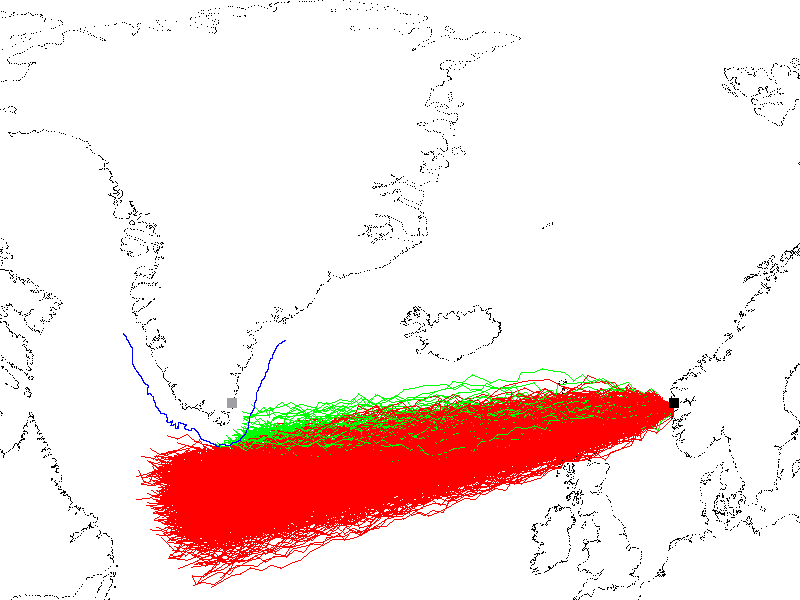


**Supplementary Figure S13.** Simulated successful (green) and unsuccessful (red) routes of 1000 Viking voyages from Bergen to Greenland at spring equinox, if a tourmaline sunstone crystal is used to analyse sky polarization with a navigation periodicity Δ*t* = 1 h, when the navigation success is 100 % (Supplementary Table S1).


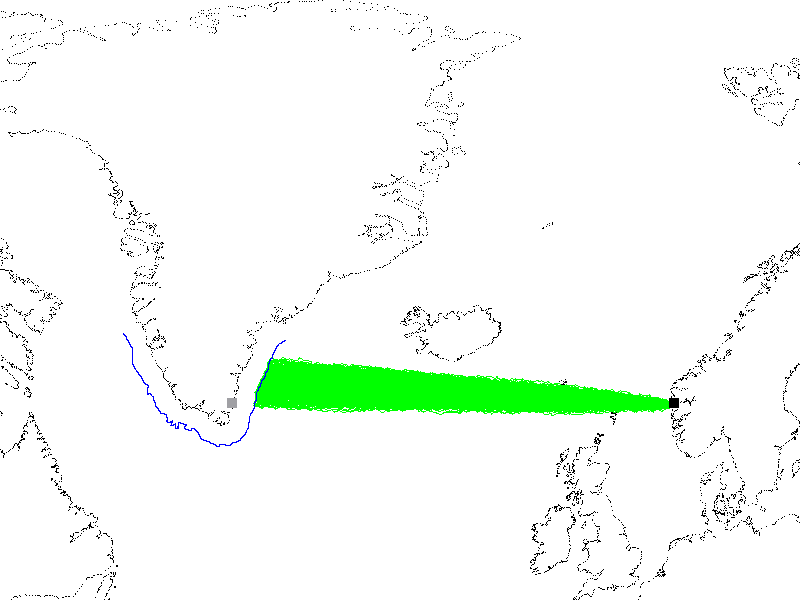


**Supplementary Figure S14.** Simulated successful (green) and unsuccessful (red) routes of 1000 Viking voyages from Bergen to Greenland at spring equinox, if a tourmaline sunstone crystal is used to analyse sky polarization with a navigation periodicity Δ*t* = 2 h, when the navigation success is 100 % (Supplementary Table S1).


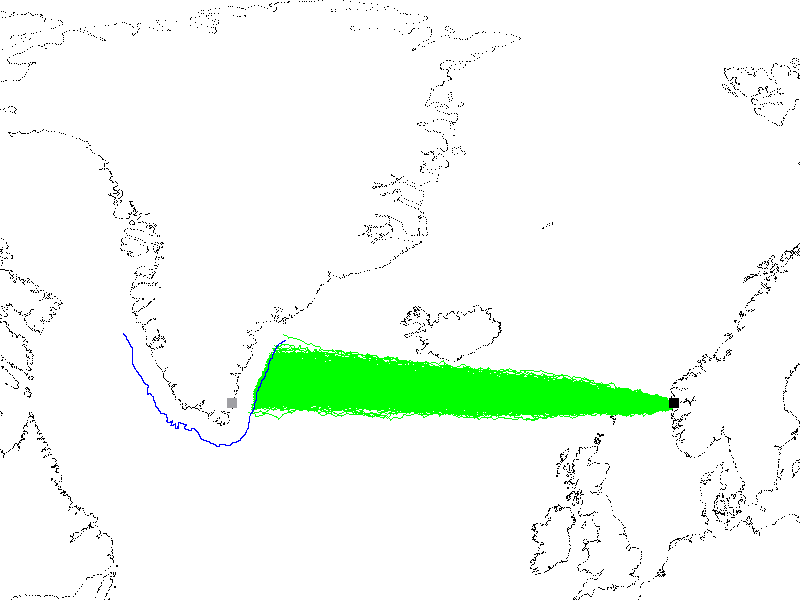


**Supplementary Figure S15.** Simulated successful (green) and unsuccessful (red) routes of 1000 Viking voyages from Bergen to Greenland at spring equinox, if a tourmaline sunstone crystal is used to analyse sky polarization with a navigation periodicity Δ*t* = 3 h, when the navigation success is 100 % (Supplementary Table S1). If some sailing trajectories went through Iceland, it was assumed that the Vikings continued their voyage toward Greenland.


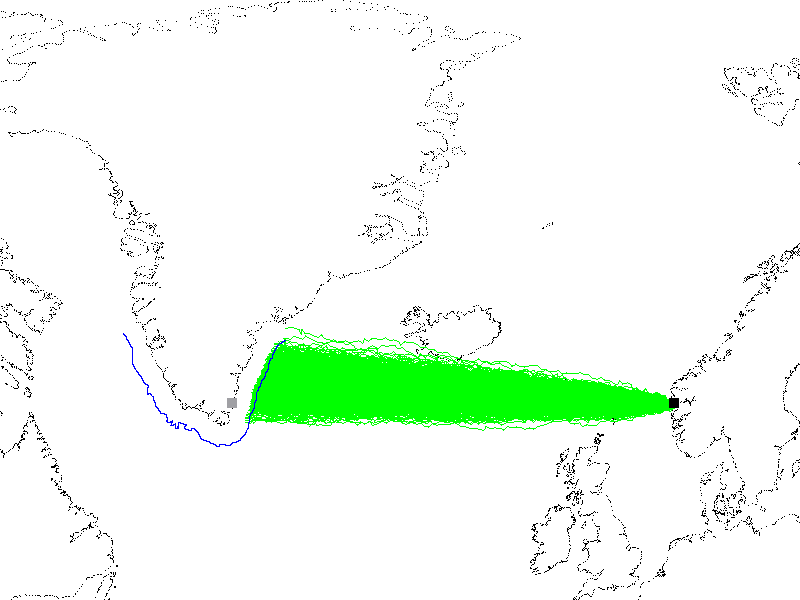


**Supplementary Figure S16.** Simulated successful (green) and unsuccessful (red) routes of 1000 Viking voyages from Bergen to Greenland at spring equinox, if a tourmaline sunstone crystal is used to analyse sky polarization with a navigation periodicity Δ*t* = 4 h, when the navigation success is 46.5 % (Supplementary Table S1).


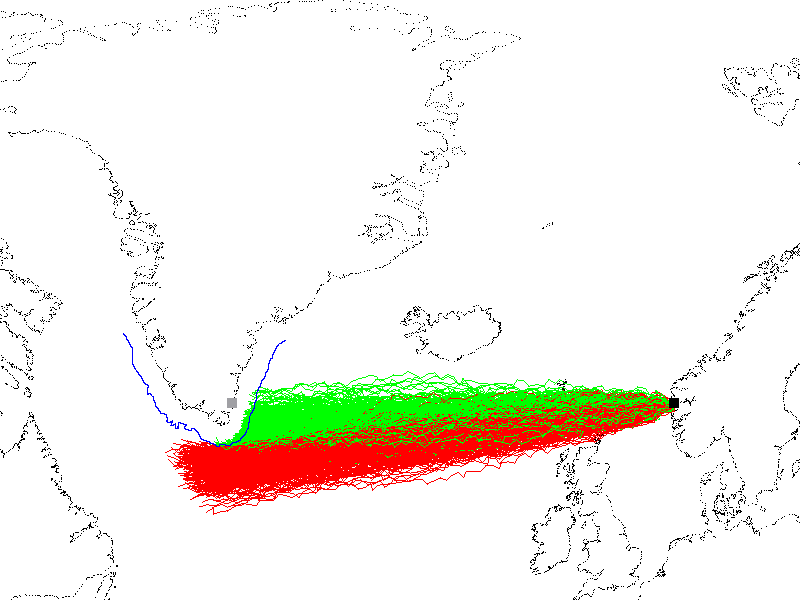


**Supplementary Figure S17.** Simulated successful (green) and unsuccessful (red) routes of 1000 Viking voyages from Bergen to Greenland at spring equinox, if a tourmaline sunstone crystal is used to analyse sky polarization with a navigation periodicity Δ*t* = 5 h, when the navigation success is 99.4 % (Supplementary Table S1). If some sailing trajectories went through Iceland, it was assumed that the Vikings continued their voyage toward Greenland.


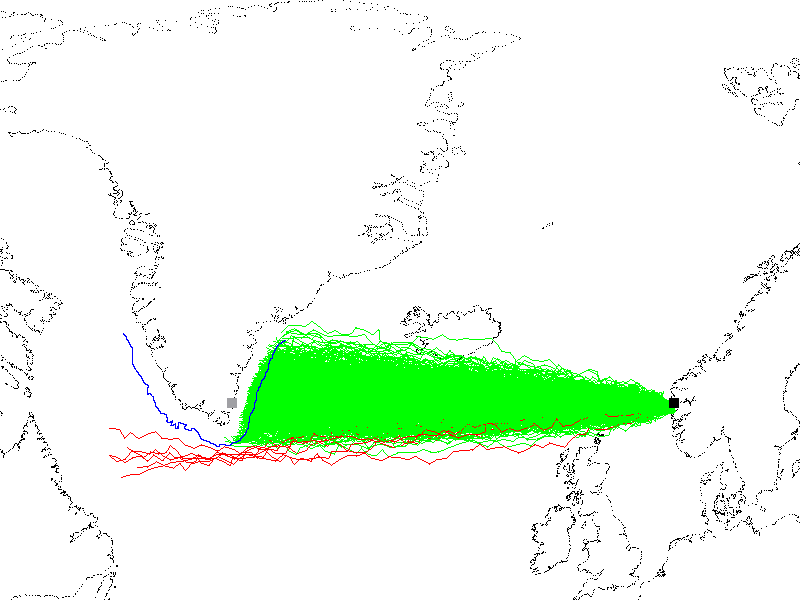


**Supplementary Figure S18.** Simulated successful (green) and unsuccessful (red) routes of 1000 Viking voyages from Bergen to Greenland at spring equinox, if a tourmaline sunstone crystal is used to analyse sky polarization with a navigation periodicity Δ*t* = 6 h, when the navigation success is 4.4 % (Supplementary Table S1). If some sailing trajectories went through North Scotland, it was assumed that the Vikings continued their voyage toward Greenland.


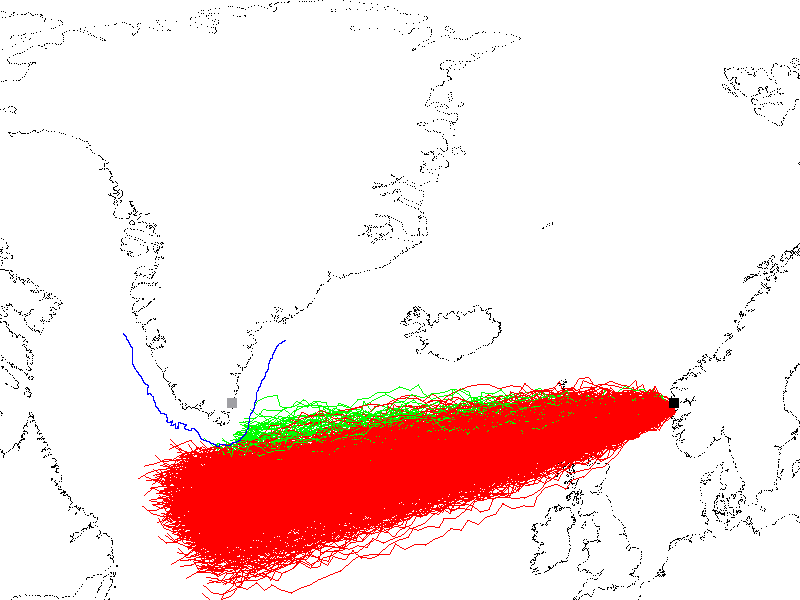


**Supplementary Figure S19.** Simulated successful (green) and unsuccessful (red) routes of 1000 Viking voyages from Bergen to Greenland at summer solstice, if a calcite sunstone crystal is used to analyse sky polarization with a navigation periodicity Δ*t* = 1 h, when the navigation success is 100 % (Supplementary Table S1).


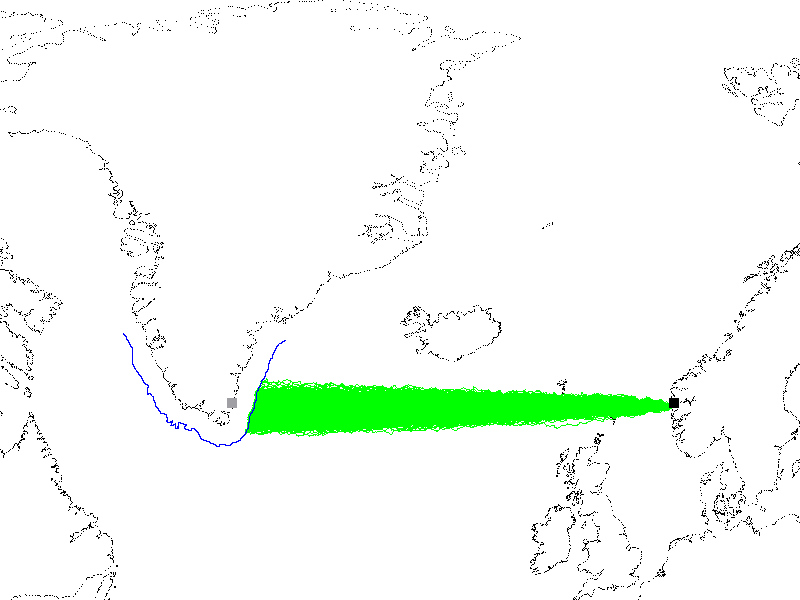


**Supplementary Figure S20.** Simulated successful (green) and unsuccessful (red) routes of 1000 Viking voyages from Bergen to Greenland at summer solstice, if a calcite sunstone crystal is used to analyse sky polarization with a navigation periodicity Δ*t* = 2 h, when the navigation success is 100 % (Supplementary Table S1).


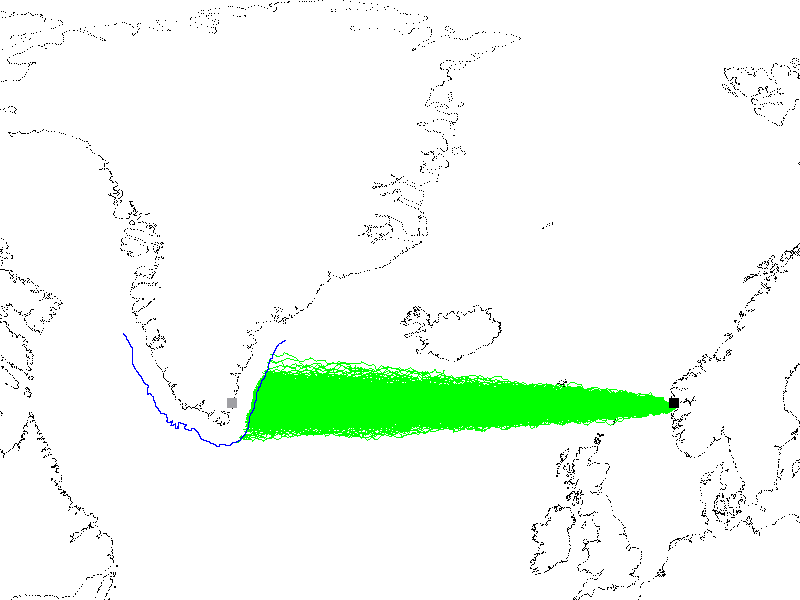


**Supplementary Figure S21.** Simulated successful (green) and unsuccessful (red) routes of 1000 Viking voyages from Bergen to Greenland at summer solstice, if a calcite sunstone crystal is used to analyse sky polarization with a navigation periodicity Δ*t* = 3 h, when the navigation success is 93.3 % (Supplementary Table S1).


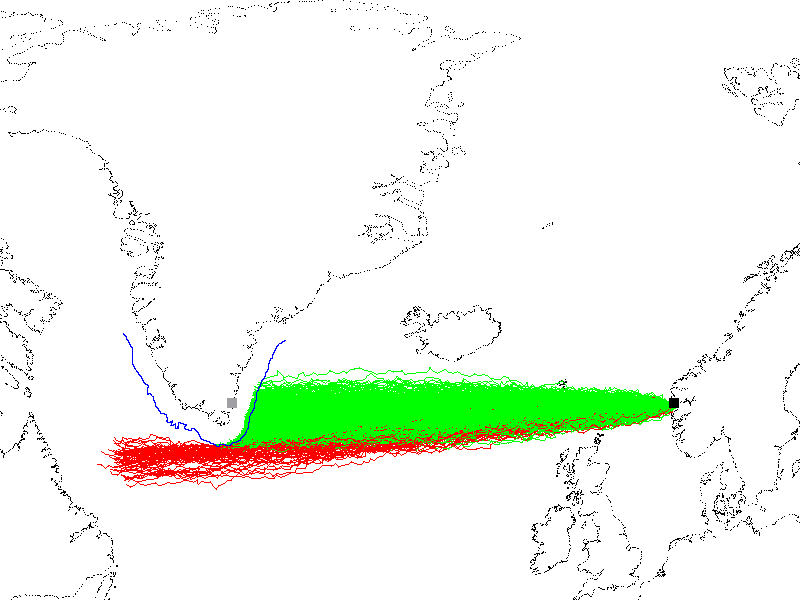


**Supplementary Figure S22.** Simulated successful (green) and unsuccessful (red) routes of 1000 Viking voyages from Bergen to Greenland at summer solstice, if a calcite sunstone crystal is used to analyse sky polarization with a navigation periodicity Δ*t* = 4 h, when the navigation success is 44.9 % (Supplementary Table S1).


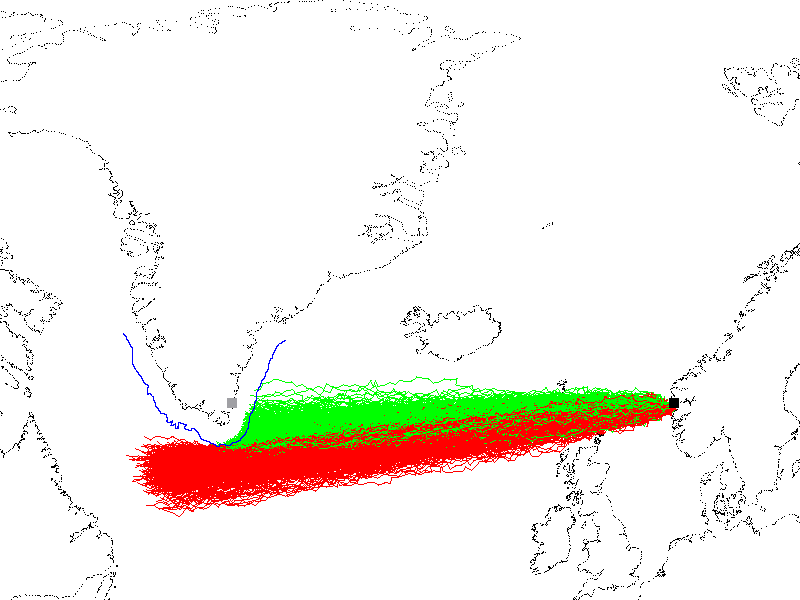


**Supplementary Figure S23.** Simulated successful (green) and unsuccessful (red) routes of 1000 Viking voyages from Bergen to Greenland at summer solstice, if a calcite sunstone crystal is used to analyse sky polarization with a navigation periodicity Δ*t* = 5 h, when the navigation success is 2.8 % (Supplementary Table S1). If some sailing trajectories went through North Scotland, it was assumed that the Vikings continued their voyage toward Greenland.


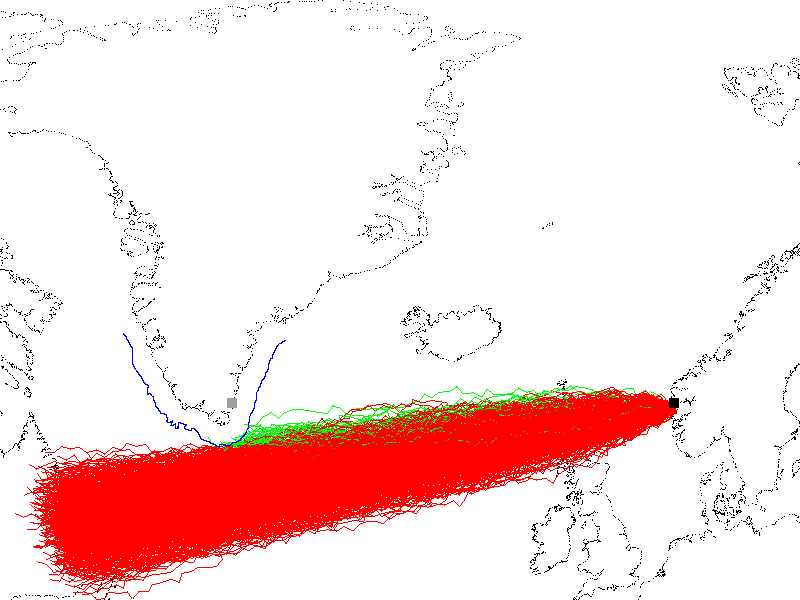


**Supplementary Figure S24.** Simulated successful (green) and unsuccessful (red) routes of 1000 Viking voyages from Bergen to Greenland at summer solstice, if a calcite sunstone crystal is used to analyse sky polarization with a navigation periodicity Δ*t* = 6 h, when the navigation success is 0.9 % (Supplementary Table S1). If some sailing trajectories went through North Scotland, it was assumed that the Vikings continued their voyage toward Greenland.


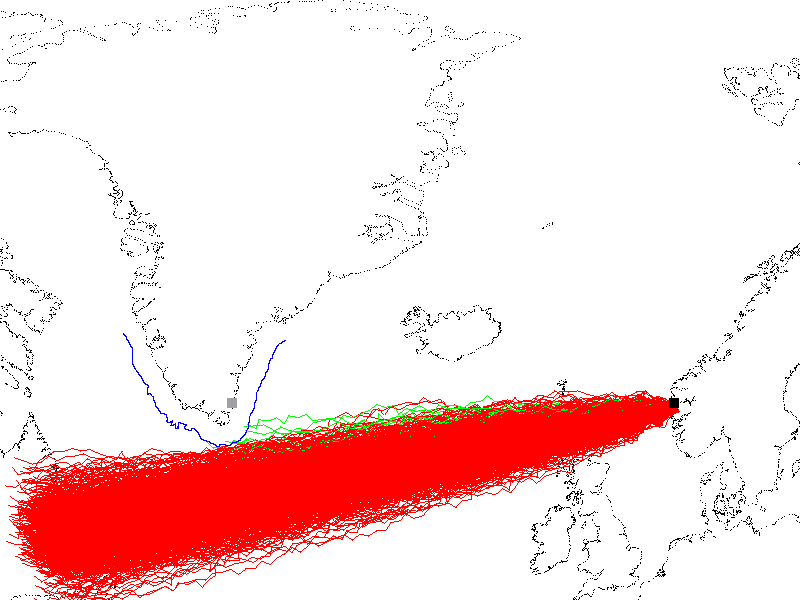


**Supplementary Figure S25.** Simulated successful (green) and unsuccessful (red) routes of 1000 Viking voyages from Bergen to Greenland at summer solstice, if cordierite sunstone crystal is used to analyse sky polarization with a navigation periodicity Δ*t* = 1 h, when the navigation success is 100 % (Supplementary Table S1).


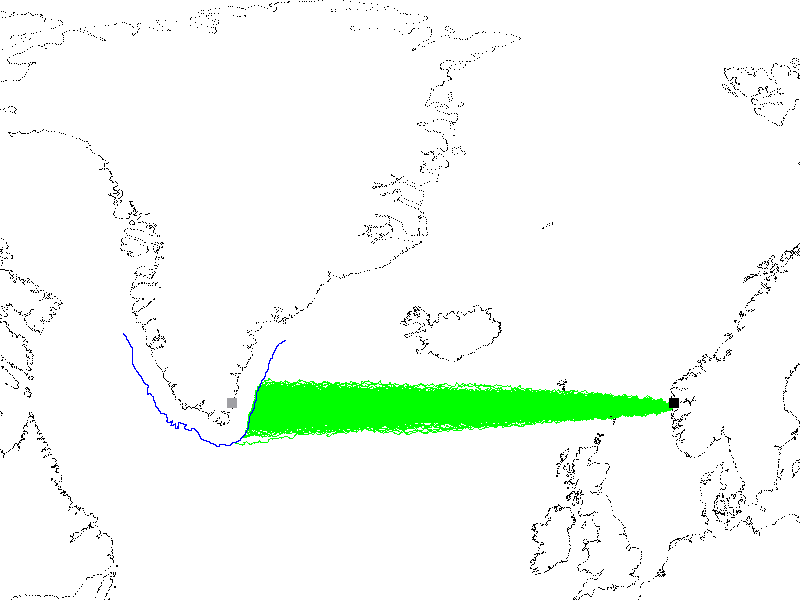


**Supplementary Figure S26.** Simulated successful (green) and unsuccessful (red) routes of 1000 Viking voyages from Bergen to Greenland at summer solstice, if cordierite sunstone crystal is used to analyse sky polarization with a navigation periodicity Δ*t* = 2 h, when the navigation success is 100 % (Supplementary Table S1).


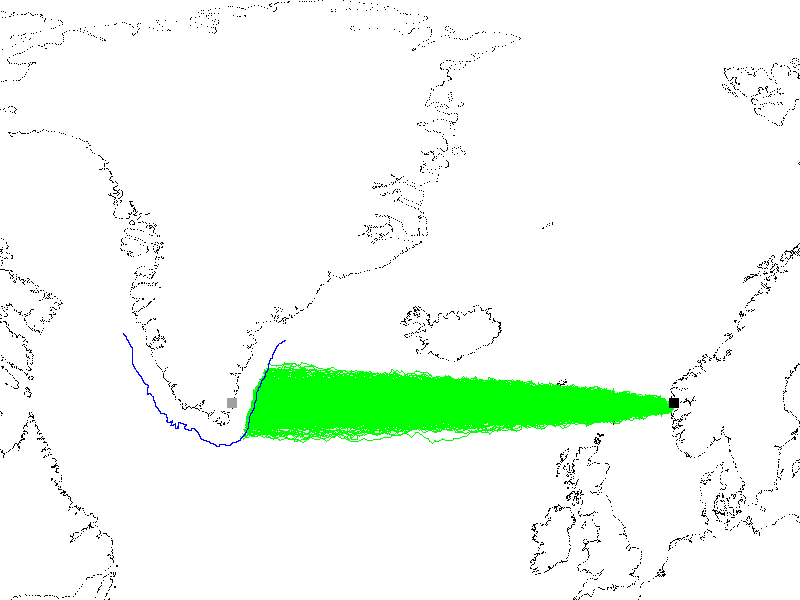


**Supplementary Figure S27.** Simulated successful (green) and unsuccessful (red) routes of 1000 Viking voyages from Bergen to Greenland at summer solstice, if cordierite sunstone crystal is used to analyse sky polarization with a navigation periodicity Δ*t* = 3 h, when the navigation success is 96.7 % (Supplementary Table S1).


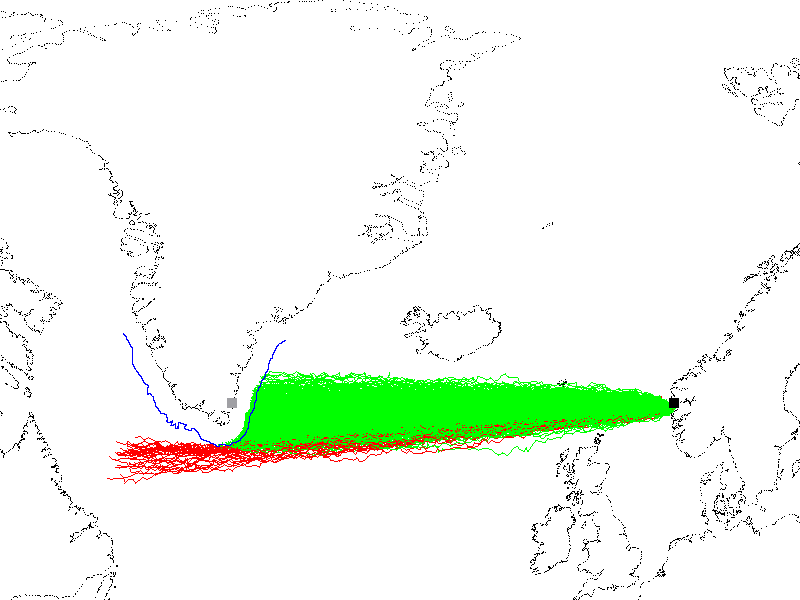


**Supplementary Figure S28.** Simulated successful (green) and unsuccessful (red) routes of 1000 Viking voyages from Bergen to Greenland at summer solstice, if cordierite sunstone crystal is used to analyse sky polarization with a navigation periodicity Δ*t* = 4 h, when the navigation success is 47.5 % (Supplementary Table S1).


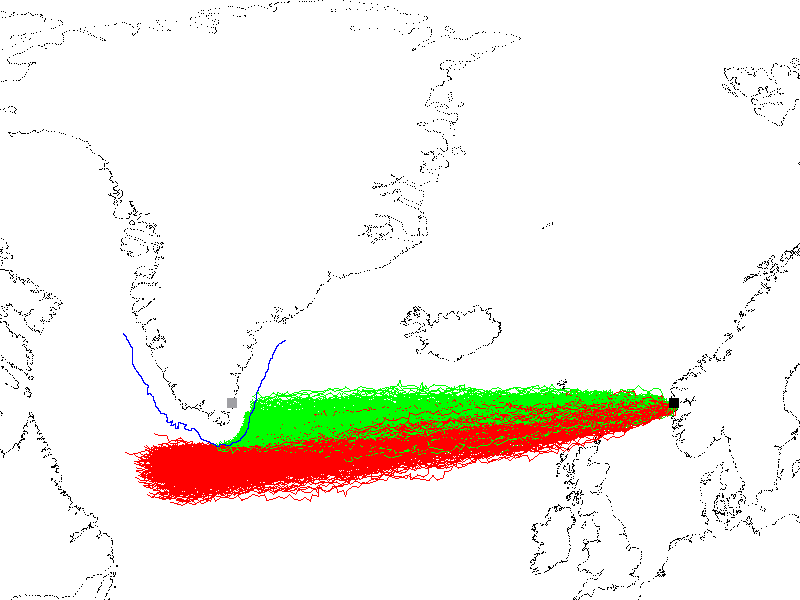


**Supplementary Figure S29.** Simulated successful (green) and unsuccessful (red) routes of 1000 Viking voyages from Bergen to Greenland at summer solstice, if cordierite sunstone crystal is used to analyse sky polarization with a navigation periodicity Δ*t* = 5 h, when the navigation success is 3.9 % (Supplementary Table S1). If some sailing trajectories went through North Scotland, it was assumed that the Vikings continued their voyage toward Greenland.


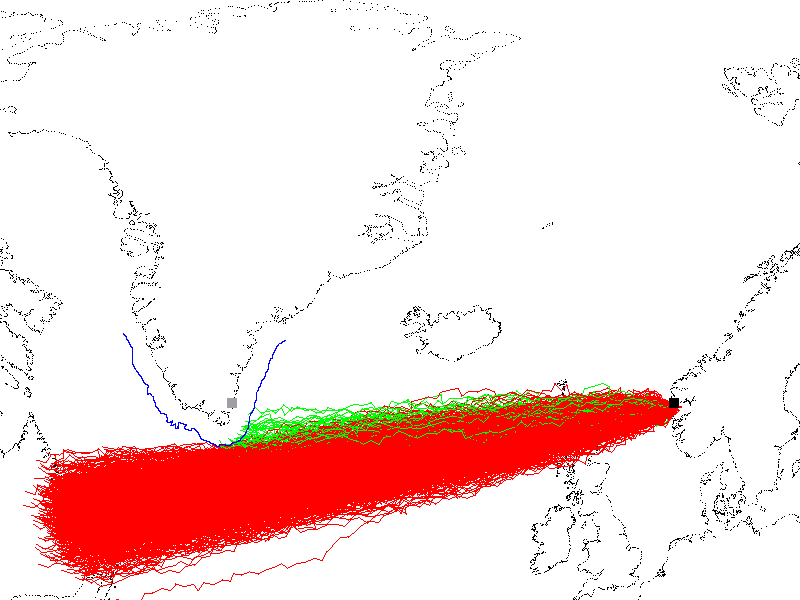


**Supplementary Figure S30.** Simulated successful (green) and unsuccessful (red) routes of 1000 Viking voyages from Bergen to Greenland at summer solstice, if cordierite sunstone crystal is used to analyse sky polarization with a navigation periodicity Δ*t* = 6 h, when the navigation success is 1.4 % (Supplementary Table S1). If some sailing trajectories went through North Scotland, it was assumed that the Vikings continued their voyage toward Greenland.


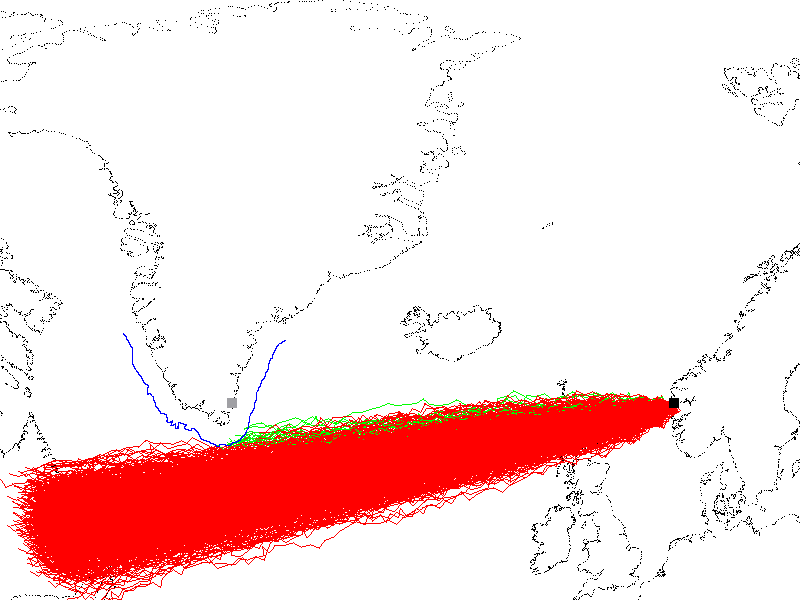


**Supplementary Figure S31.** Simulated successful (green) and unsuccessful (red) routes of 1000 Viking voyages from Bergen to Greenland at summer solstice, if tourmaline sunstone crystal is used to analyse sky polarization with a navigation periodicity Δ*t* = 1 h, when the navigation success is 100 % (Supplementary Table S1).


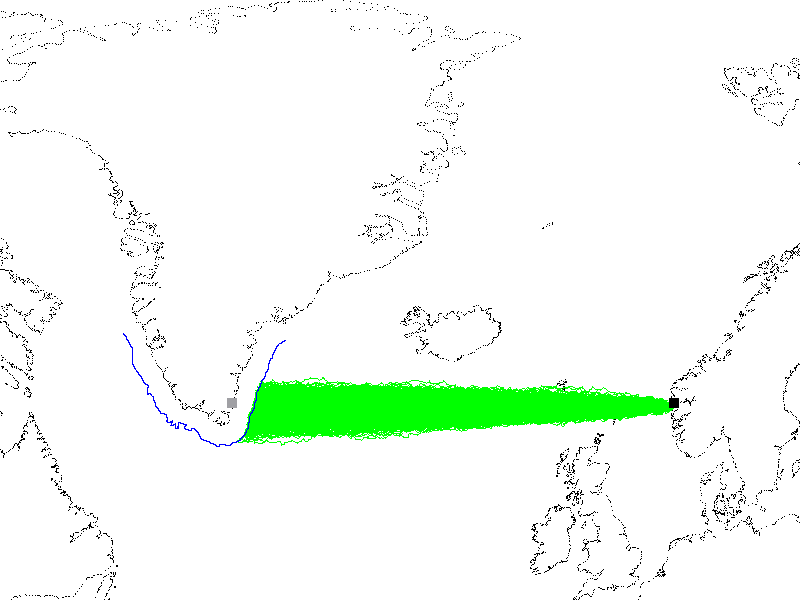


**Supplementary Figure S32.** Simulated successful (green) and unsuccessful (red) routes of 1000 Viking voyages from Bergen to Greenland at summer solstice, if tourmaline sunstone crystal is used to analyse sky polarization with a navigation periodicity Δ*t* = 2 h, when the navigation success is 99.9 % (Supplementary Table S1).


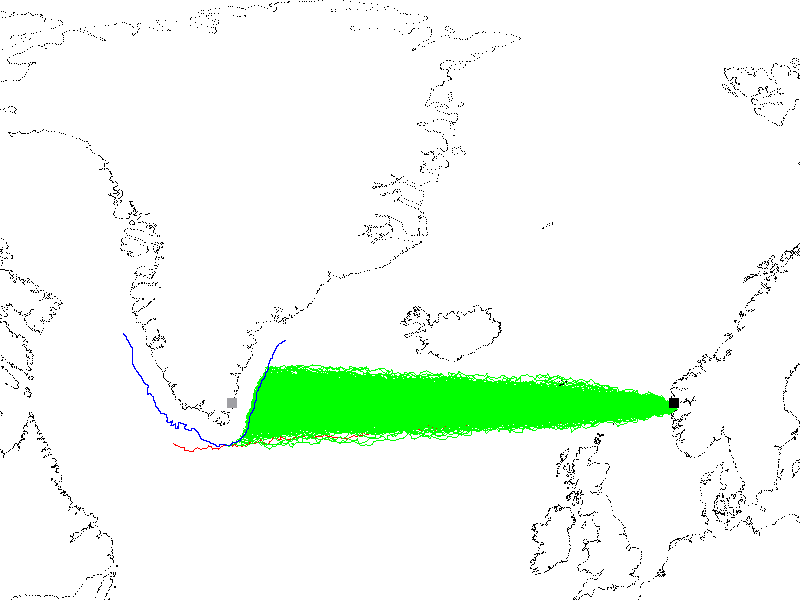


**Supplementary Figure S33.** Simulated successful (green) and unsuccessful (red) routes of 1000 Viking voyages from Bergen to Greenland at summer solstice, if tourmaline sunstone crystal is used to analyse sky polarization with a navigation periodicity Δ*t* = 3 h, when the navigation success is 92.2 % (Supplementary Table S1).


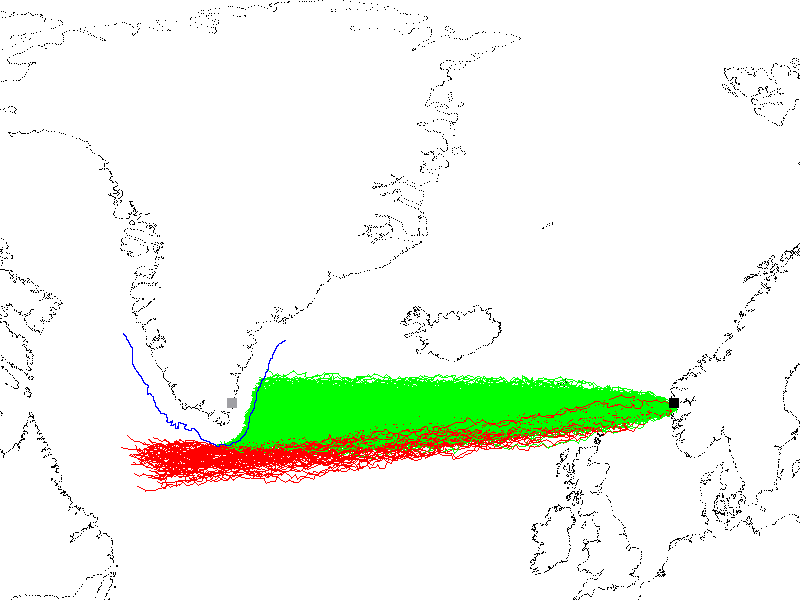


**Supplementary Figure S34.** Simulated successful (green) and unsuccessful (red) routes of 1000 Viking voyages from Bergen to Greenland at summer solstice, if tourmaline sunstone crystal is used to analyse sky polarization with a navigation periodicity Δ*t* = 4 h, when the navigation success is 32.1 % (Supplementary Table S1). If some sailing trajectories went through North Scotland, it was assumed that the Vikings continued their voyage toward Greenland.


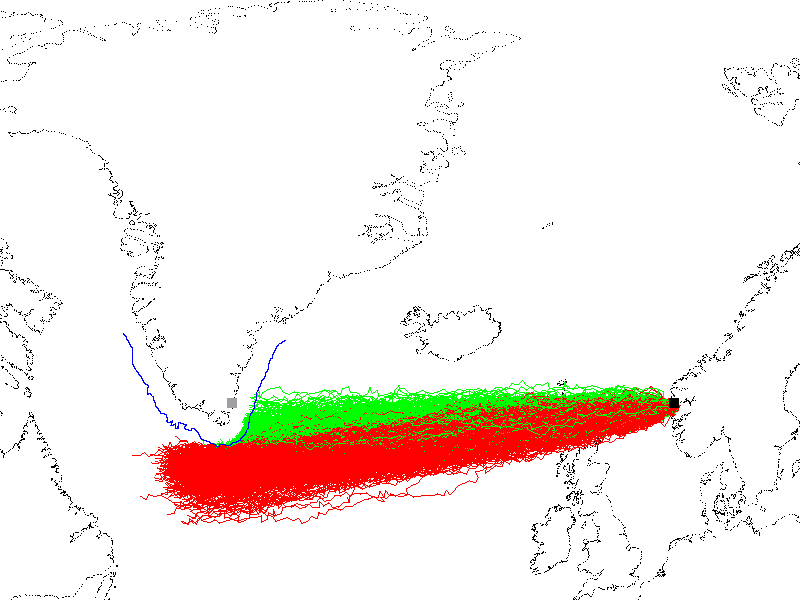


**Supplementary Figure S35.** Simulated successful (green) and unsuccessful (red) routes of 1000 Viking voyages from Bergen to Greenland at summer solstice, if tourmaline sunstone crystal is used to analyse sky polarization with a navigation periodicity Δ*t* = 5 h, when the navigation success is 4.0 % (Supplementary Table S1). If some sailing trajectories went through North Scotland, it was assumed that the Vikings continued their voyage toward Greenland.


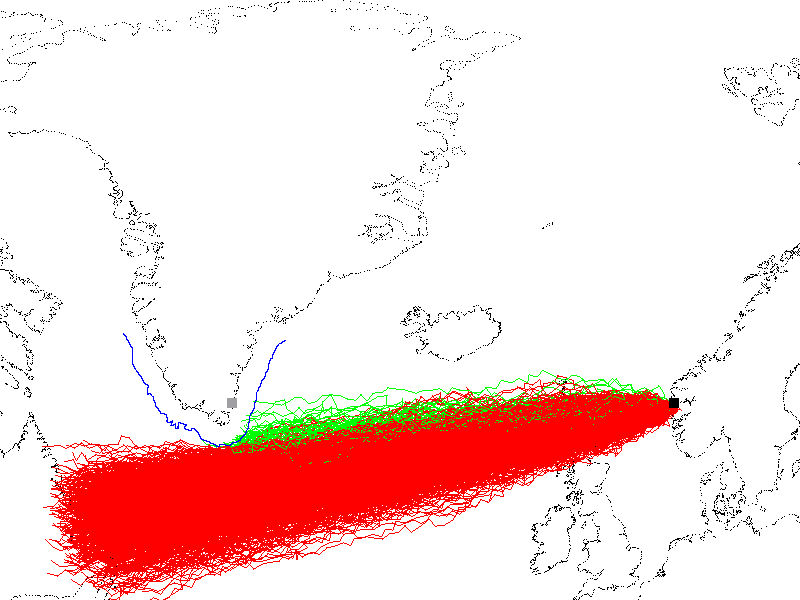


**Supplementary Figure S36.** Simulated successful (green) and unsuccessful (red) routes of 1000 Viking voyages from Bergen to Greenland at summer solstice, if tourmaline sunstone crystal is used to analyse sky polarization with a navigation periodicity Δ*t* = 6 h, when the navigation success is 1.9 % (Supplementary Table S1). If some sailing trajectories went through North Scotland, it was assumed that the Vikings continued their voyage toward Greenland.


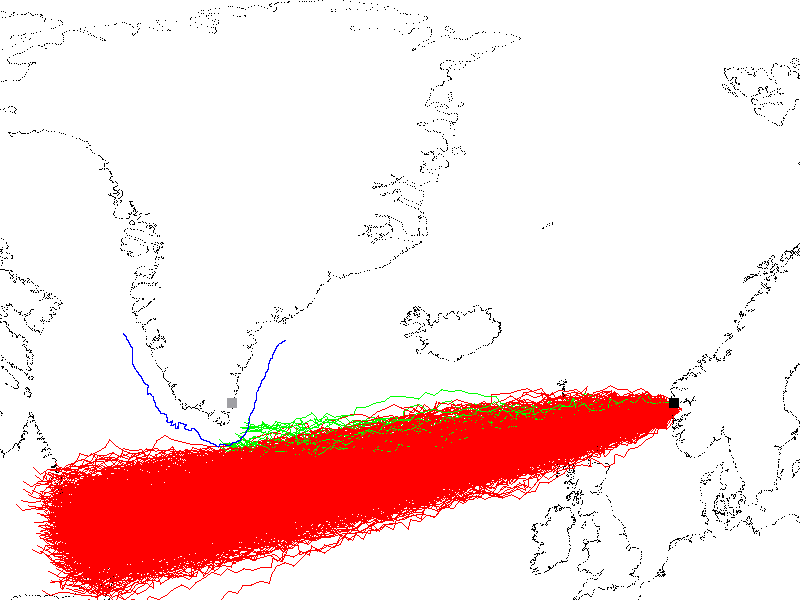


**Supplementary Figure S37.** Geometry of the curving Earth’s surface near the coast of Greenland to calculate the borderline of visibility of the mountains of Greenland.*r*: Earth’s radius, *m*: average mountain height, *c*: distance of the mountain from the coast C, *h*: height of the observer on the ship’s mast M measured from the sea level, α: angular distance of the point P at which the tangential straight line from the mountain top T touches the Earth's surface, β: angular distance between points P and M, *d*: distance (measured from C) of the border from where the mountain top T can already be seen from M. The shape of the Earth is approximated by a sphere of radius *r*. The sizes are not proportional for the sake of a better visualization.


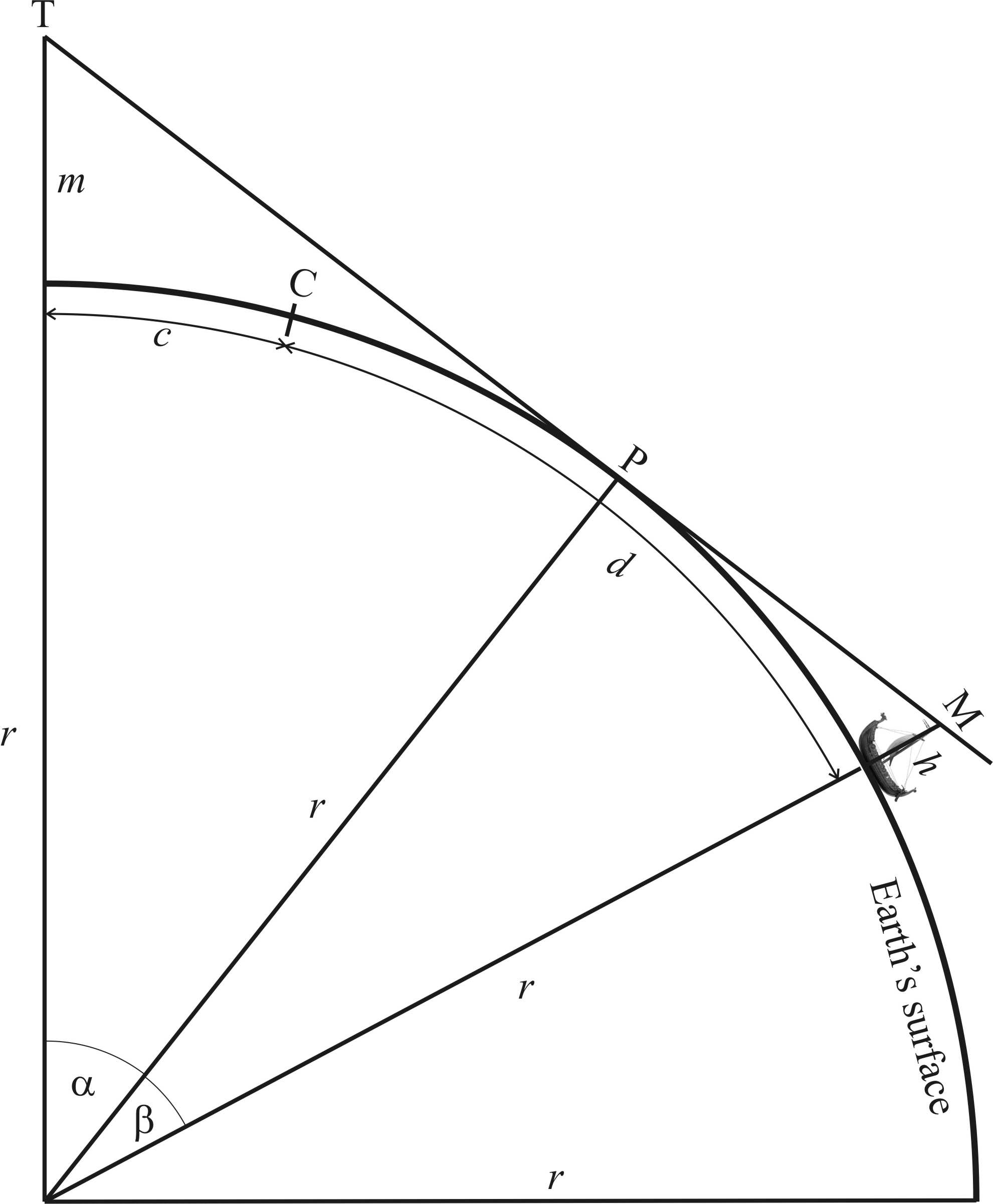

Supplement: Supplementary Materials [file rsos172187supp1.docx]
